# Effects of exercise training on frail older adults with heart failure: a systematic-review

**DOI:** 10.3389/fragi.2026.1800669

**Published:** 2026-03-26

**Authors:** C. Sáez-Nieto, P. Pérez-Rodríguez, P. Matovelle, L. Rodríguez-Mañas, H. J. Coelho-Júnior, I. Rodríguez-Sánchez

**Affiliations:** 1 Geriatrics Department, Hospital Universitario Infanta Leonor, Madrid, Spain; 2 Geriatrics Department, Hospital Universitario Puerta de Hierro, Majadahonda, Madrid, Spain; 3 Geriatrician, Hospital Monte Sinai, Cuenca, Ecuador; 4 Geriatrics Department, Hospital Universitario de Getafe, Madrid, Spain; 5 Centro de Investigación Biomédica en Red Fragilidad y Envejecimiento Saludable (CIBERFES), Madrid, Spain; 6 Fondazione Policlinico Universitario Agostino Gemelli, IRCCS, Rome, Italy; 7 Department of Geriatrics and Orthopedics, Università Cattolica del Sacro Cuore, Rome, Italy; 8 Geriatrics Department, Hospital Universitario Clínico San Carlos, Madrid, Spain; 9 Fundación para la Investigación Biomédica del Hospital Clínico San Carlos (IdISSC), Madrid, Spain; 10 Universidad Complutense de Madrid, Madrid, Spain

**Keywords:** cardiac rehabilitation, exercise training, frailty, heart failure, older adults, physical function, systematic review

## Abstract

**Introduction and objectives:**

Heart failure (HF) is highly prevalent in older adults and is frequently associated with frailty, leading to increased morbidity, hospitalization, disability, and mortality. Exercise training (ET) has demonstrated benefits in HF and frailty separately, but its effects in frail older adults with HF have not been extensively evaluated. This systematic review aimed to synthesize the evidence on the effects of ET on health outcomes in frail older patients with HF.

**Methods:**

A systematic review of interventional studies was conducted following PRISMA and Cochrane Handbook guidelines. MEDLINE (PubMed), SCOPUS, and Scielo were searched up to March 2026. Inclusion criteria were: (a) intervention studies involving frail adults aged ≥60 years with HF, (b) evaluation of chronic effects of ET on health outcomes, and (c) publication in English. Study selection and data extraction were performed independently by four reviewers, with a fifth reviewer resolving disagreements. Methodological quality of randomized controlled trials was assessed using the PEDro scale.

**Results:**

Six investigations were included. Overall quality assessment results ranged from 4 to 7. Studies were conducted in Japan and the United States. All were randomized controlled trials with sample sizes ranging from 30 to 337 participants (mean age 72.5 years). Frailty was assessed using the Frailty Phenotype, Frailty Index, and Short Physical Performance Battery. ET interventions varied in duration and modality, and were mostly characterized by multicomponent (e.g., resistance, endurance, flexibility) exercise training protocols. Adverse events were infrequent; one study reported musculoskeletal pain in 25% of participants. ET significantly improved HF symptoms, frailty status, physical function (mobility, muscle strength, aerobic capacity), physical activity levels, quality of life, and depressive symptoms. Improvements in hemoglobin and cholesterol were also observed. Meta-analysis was not performed due to heterogeneity in interventions and outcome measures.

**Conclusion:**

Cardiac rehabilitation programs based on ET protocols appear to improve clinical and functional outcomes in frail older adults with HF. However, evidence is limited by the small number of studies, variability in intervention protocols, and heterogeneity in outcome assessment. Further high-quality randomized trials are needed to confirm these findings and establish optimal ET strategies for this vulnerable population.

## 1 Introduction

World Health Organization projections indicate that individuals aged 60+ years will reach nearly two billion people by 2050 worldwide, suppressing the young population. Although this demographic phenomenon represents decades of advances in medical care, the aging process is associated with the development of chronic conditions (e.g., cardiovascular diseases), which characterize an important challenge for health systems (James et al., 2018; Salomon et al., 2012). For instance, a significant reduction in physiological reserve accompanied by an increased vulnerability to stressors events is frequently observed in people with advanced age, a condition called frailt y (Fried et al., 2001).

Frailty is acknowledged as a geriatric syndrome that contributes to the development of diseases and increases the risk for many adverse outcomes, including falls, sarcopenia, hospitalization, disability, institutionalization, and death (Fried et al., 2001; Morley et al., 2013; Rodríguez-Mañas et al., 2013; Afilalo et al., 2014). Furthermore, several studies have shown that frailty represents a stronger predictor of these negative events than other well-established conditions (e.g., multimorbidity, sarcopenia) (Davies et al., 2022).

Another prevalent condition observed in people with advanced age is heart failure (HF) (Khan et al., 2024). Current estimations indicate that nearly 2 in each 10 old individuals have HF (Khan et al., 2024). These data are concerning, as HF is a major cause of poor quality of life, functional decline, hospitalization, and death among older people, reflecting in exacerbated healthcare costs (Khan et al., 2024; Darbà et al., 2025). Notably, numerous studies have found that HF commonly coexist with frailty (Denfeld et al., 2017).

Indeed, frailty is very common in older adults with HF, affecting nearly 80% of this population, according to individuals’ characteristics (Altimir et al., 2005; Lupón et al., 2008; McNallan et al., 2013; Denfeld et al., 2017). This relationship has serious health implications (Veronese et al., 2017; Yang et al., 2018; Prokopidis et al., 2025), with studies indicating numerous negative outcomes, including reducing exercise tolerance (Kato, 2013; Springer et al., 2017; Suzuki et al., 2018; Yin et al., 2019), increased risk of hospital admission (Chaudhry et al., 2010; Vidán et al., 2016), and death (Vidán et al., 2016; Yang et al., 2018). As such, the prompt implementation of therapeutic strategies to avoid the progression and harmful consequences of frailty among people with HF is mandatory to preserve the quality of life and independence of these individuals.

Exercise training (ET) is globally acknowledged as a first-line therapy to counteract frailty (Hoogendijk et al., 2019), with studies supporting its effectiveness in frail older adults from different contexts (Nagata et al., 2023; Yang et al., 2024). Similarly, ET is a cardinal strategy to contribute to the rehabilitation of HF patients. In fact, international organizations responsible for the care of HF patients have instructed the use of ET to maintain or even improve multiple health aspects in individuals with this condition. These recommendations are grounded in the numerous randomized controlled trials (RCT) that showed improvements in cardiac function (e.g., ejection fraction), physical performance, and quality of life, among other domains. Investigations have also examined the effects of ET on HF patients with frailty (Papathanasiou, 2020; Pandey et al., 2023). However, to the best of our knowledge, no systematic reviews have been conducted so far.

Based on these premises, the present study aimed at investigating the effects of ET on health-aspects of older adults with HF and frailty.

## 2 Methods

We conducted a systematic review of interventional studies to quantify the effects of ET on frail older adults with HF. The study was fully performed by investigators, and no librarian was part of the team. This study complies with the criteria of the Primary Reporting Items for Systematic Reviews and Meta-Analyses (PRISMA) Statement (Liberati et al., 2009) and Cochrane Handbook for Systematic Reviews and Interventions (Green and Higgins, 2005). All data are available in the open Science Framework (https://osf.io/c25nj).

### 2.1 Eligibility criteria

The inclusion criteria of the present study consisted of: (a) investigations that studied the chronic effects (≥4 weeks) of ET protocols on any health parameter; (b) examined frail older adults with HF aged ≥60 years; (c) published studies in English language. Studies analyzing exclusive nutritional or pharmacological strategies were excluded. We also excluded studies examining multidomain strategies where ET was not the main intervention. No limitations were set regarding the frailty assessment tools used.

### 2.2 Search strategy and selection criteria

Studies published on or before March 2026 were retrieved by five investigators from MEDLINE (PubMed interface), SCOPUS, and SciELO. Reference lists for reviews and retrieved articles for additional studies were checked and citation searches in key articles were performed on Google Scholar and ResearchGate for additional reports. Initially, a search strategy was designed using keywords such as “exercise”, “frail older adults”, and “heart failure”. Additionally, keywords and subject headings were exhaustively combined using Boolean operators. Only eligible full texts in English and Spanish were considered for review.

### 2.3 Data extraction and quality assessment

Titles and abstracts of retrieved articles were screened for eligibility by four researchers. Full-text articles were retrieved when abstracts did not provide sufficient information for evaluation. Four reviewers independently extracted study characteristics and methodological data using a standardized coding form. A fifth researcher was consulted to resolve any disagreements.

The methodological quality of each included intervention trial was assessed using the PEDro scale (de Morton, 2009). This assessment tool evaluates key aspects of trial design and reporting, including random and concealed allocation, baseline comparability, blinding, follow-up adequacy, intention-to-treat analysis, and reporting of between-group statistical comparisons. Four reviewers performed the assessment independently, and discrepancies were resolved by discussion or consultation with a fifth researcher. The inter-rater agreement for PEDro scoring was high (κ = 0.99).

## 3 Results

### 3.1 Search results

**FIGURE 1:**
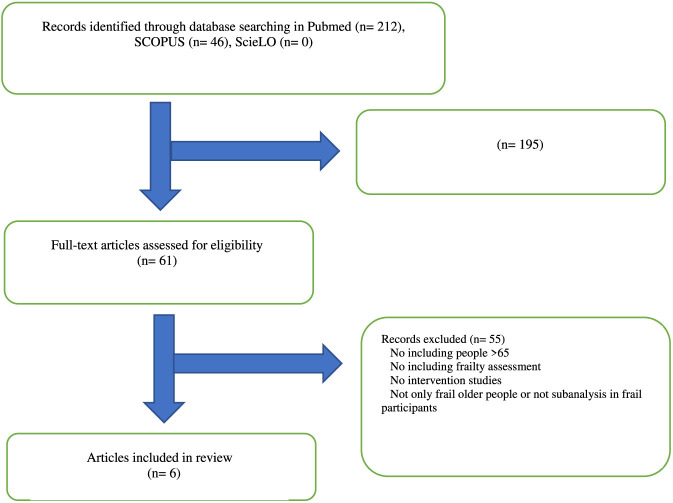
Selection process of studies.

A detailed summary of the literature search is provided in Figure 1. A total of 258 records were retrieved: 212 from PubMed and hand searching and 46 from Scopus. No records were retrieved from SciELO. Of these, 195 records were excluded after screening titles and abstracts. Two records were identified in both PubMed and Scopus and were therefore merged. Sixty-one full-text articles were assessed for eligibility, and 55 were excluded, leaving a total of six articles (n = 708 participants).

### 3.2 Participants’ characteristics

The main characteristics of the studies included are shown in Table 1. All the studies included were RCTs. Investigations were published from 2005 to 2023, and conducted in the United States of America (n = 2), Japan (n = 1), Australia (n = 1), Bulgaria (n = 1), and Spain (n = 1). Nearly 700 participants (n = 708) with a mean age of 72.5 years were examined. Frailty was identified using original and modified versions of the Frailty Phenotype (n = 2), the Frailty Index (n = 1), and the Short Physical Performance Battery (SPPB) (n = 1). Overall quality assessment results ranged from 4 to 7. The incidence of adverse events during exercise interventions across the studies was generally low, with most interventions proving to be safe and well-tolerated by participants.

**TABLE 1:** Main characteristics of the study participants of the included studies.

First author	Year	Country	Design	Sample size	Mean participants’ age	Frailty assessment method	Intervention	Quality assessment (0–10)	Results
Dobarro D.	2023	Spain	RCT single-blinded	48	83.0	SPPB	VIVIFRAIL	6	↑ HF symptoms; ↑ PA
Pandey A.	2023	United States	RCT	337	72.0	Frailty phenotype	Progressive multidomain physical rehabilitation intervention	5	↑ SPPB, ↑ Aerobic capacity; ↑ Mobility, ↑ Muscle strength; ↑ QoL, ↓ Depressive symptoms
Nagatomi Y.	2022	Japan	RCT	30	63.7	Japanese cardiovascular health study	Comprehensive cardiac rehabilitation program focused on exercise training	4	↑ Aerobic capacity and mobility; ↑ Muscle strength; ↑ PA; ↑ Hemoglobin; ↑ Cholesterol
Mudge A. M.	2021	Australia	RCT	91	>70	Frailty index	Multicomponent exercise training	5	↑ Frailty index
Papathanasiou J. V.	2020	Bulgaria	RCT	120	63.7	—	Moderate and HIIT	5	↑ Aerobic capacity; ↑ Mobility; ↑ QoL
Witham M. D.	2005	United States	RCT	82	80.0	—	Exercise training	7	↑ PA; ↓ Depressive symptoms

HF, heart failure; HIIT, High-intensity interval training; PA, physical activity; QoL = quality of life; RCT, randomized controlled trial; SPPB, Short Physical Performance Battery. ↑ = Improvement; ↓ = Reduction.

### 3.3 Effects of exercise intervention on symptom-limited exercise tolerance

The effect of ET on exercise tolerance was examined in one study. Dobarro et al. (Dobarro et al., 2023) investigated the effects of a 6-month VIVIFRAIL program on the severity of HF symptoms. The authors found that 47.6% of participants in the ET program showed improvements of at least one point in the New York Heart Association (NYHA) classification, a gold-standard instrument to assess functional limitation due to HF, compared to only 22.2% in the control group.

### 3.4 Effects of exercise intervention on frailty status

The effect of ET on frailty status was examined in one study. Mudge et al. (Mudge et al., 2021) proposed a 6-month ET-based cardiac rehabilitation program composed of both home- and center-based resistance, aerobic, and balance exercises. Participants of the intervention group exhibited significant improvements (∼60%) in the results of the frailty index, compared to 20% in the non-exercised group.

### 3.5 Effects of exercise intervention on physical performance

The effect of ET on physical performance was investigated in three studies. Papathanasiou (Papathanasiou, 2020) investigated the effects of a group-based intervention on the aerobic capacity and mobility of frail patients with HF. The ET involved moderate- and high-intensity interval training (HIIT) protocols, performed 5 times a week, over 12 weeks. A significant 20%–30% and 11%–17% increase in aerobic capacity and mobility, respectively, were observed after interventions. These findings were expanded by Nagatomi et al. (Nagatomi et al., 2022), after investigating the effects of a home-based cardiac rehabilitation involving stretching, resistance, and aerobic exercises. Authors found significant improvements in mobility and aerobic capacity—assessed using the 6-min walking test (6MWT) — and lower-limb muscle strength. More recently, Pandey et al. (Pandey et al., 2023) conducted a secondary analysis of the REHAB-HF trial. The intervention encompassed 36 sessions over 12 weeks and focused on strength, balance, mobility, and endurance exercises. Results indicated a significant improvement of nearly 80% in SPPB results among frailty participants, in contrast to an 18% increase in the control group. Furthermore, significant increases in 6MWT, walking speed, lower-limb muscle strength, and balance were observed.

### 3.6 Effects of exercise intervention on physical activity levels

The effects of ET on PA were investigated in three studies. Witham et al. (Witham et al., 2005) proposed a two-phase 6-month ET protocol, which involved a 3-month supervised program and a 3-month home-based intervention. Authors found significant improvements in everyday PA levels, evaluated using a validated accelerometer. Findings of Nagatomi et al. (Nagatomi et al., 2022) are in line with these results, as a significant increase of nearly 700 steps/day was noted after ET. Significant increases were also observed when PA was estimated using self-reported instruments. Indeed, Dobarro et al. (Dobarro et al., 2023) reported a 6.4 improvement in the physical activity scale for the elderly (PASE) score in the intervention group, compared to a reduction of 12.5 in the control group.

### 3.7 Effects of exercise intervention on secondary outcomes

Secondary outcomes included QoL, depressive symptoms, hemoglobin, and cholesterol. Significant improvements in QoL and depressive symptomatology were observed in two studies (Papathanasiou, 2020; Pandey et al., 2023). Additionally, Nagatomi et al. (Nagatomi et al., 2022) found that ET increased cholesterol and hemoglobin levels.

## 4 Discussion

The main findings of the present study indicate that cardiac rehabilitation programs based on ET protocols significantly improved health aspects in frailty patients with HF. Specifically, symptom-limited exercise tolerance, frailty severity, and depressive symptoms were reduced, whereas the performance in multiple physical function tests (i.e., mobility, lower-limb muscle strength, aerobic capacity), PA levels, and QoL were significantly increased. Furthermore, changes in hemoglobin and cholesterol levels were observed.

These results underscore the importance of ET as a complementary strategy to pharmacological treatment in the recovery and improvement of health status in frail HF patients. Specifically, while these findings need further confirmation in additional studies, the observation that ET improved HF severity in approximately 50% of frail older adults who participated in the program (Dobarro et al., 2023) is promising and encouraging. Indeed, the fact that increased HF severity is independently associated with QoL, multimorbidity, malnutrition, major clinical events (e.g., hospitalization), and all-cause and cause-specific mortality (Liang et al., 2011; Demant et al., 2014; Agra Bermejo et al., 2017; Shuvy et al., 2020), suggests that ET may serve as a valuable tool to help reduce the overall burden of frail older adults with HF.

These premises regarding the usability of ET as a strategy to reduce HF and frailty burden in older adults are further supported by our observations on the safety and tolerability of the exercise protocols. Most intervention trials, including the one involving a HIIT protocol, did not report significant adverse events related to exercise. These findings likely reflect the efforts of study groups to align their intervention programs with established ET principles (e.g., overload, individualization) and to integrate trained movement therapists (e.g., exercise physiologists, physiotherapists) into the intervention design and supervision processes (Kraemer and Ratamess, 2004; American College of Sports Medicine, 2009). For instance, the HIIT protocol was supervised and conducted in a specialized center for rehabilitation using validated tools to monitor exercise intensity and progression (Papathanasiou, 2020).

Another important finding of the present study is that ET significantly improved frailty status and physical function. These observations have clinical implications because frailty, sarcopenia, and physical impairments are highly prevalent among patients with HF compared to the general population (Pandey et al., 2019; Zhang et al., 2021) and exacerbate the risk of adverse outcomes in this population (Pandey et al., 2019; Zhang et al., 2021). For instance, frailty may contribute to the worsening of cardiac cachexia, a condition that further complicates the management of heart failure (Lavie et al., 2014).

Furthermore, frailty, sarcopenia, and physical impairments are independent risk factors for a wide range of adverse outcomes in the older population, such as falls, fractures, institutionalization, disability, dementia, hospitalization, and death (Studenski et al., 2011; Hirani et al., 2015; Kojima, 2016; 2017b, 2017a; 2018b, 2018a; Beeri et al., 2021). These conditions have also been consistently linked to cognitive decline, multimorbidity, and polypharmacy (Auyeung et al., 2008; Huang et al., 2016; Coelho-Júnior et al., 2018; Bai et al., 2020; Beeri et al., 2021; Dai et al., 2023), further complicating the health of older adults and reinforcing the importance of ET. This complex scenario often leads to the need for increased healthcare interventions, culminating in high healthcare costs (Janssen et al., 2004; García-Nogueras et al., 2017; Hajek et al., 2018).

The practice of PA, simultaneously with ET and pharmacological treatment, is important in patients with HF because it enhances cardiovascular function, improves muscle strength, and increases exercise tolerance, which can help reduce symptoms like fatigue and dyspnea (LaMonte, 2018; Jordan et al., 2023). This combined approach helps mitigate the detrimental effects of sedentary behavior, which is common in HF patients and contributes to physical deconditioning, frailty, and poor prognosis (LaMonte, 2018; Jordan et al., 2023). Furthermore, engaging in regular PA alongside pharmacological treatment can improve overall functional capacity, reduce hospitalizations, and enhance QoL, addressing both the physical and psychological challenges of living with heart failure (LaMonte, 2018; Jordan et al., 2023).

The combination of the significant improvements in physical function, frailty status, and HF symptomatology likely explains the changes in QoL and depressive symptomatology observed in the present study. Improved physical function and reduced frailty contribute to increased independence and a greater sense of wellbeing, which directly impacts mental health and reduces feelings of depression. Furthermore, the reduction in HF symptoms, such as breathlessness and fatigue, enables patients to engage more actively in daily activities, leading to a better QoL. The positive impact on physical health can therefore foster a more optimistic outlook, helping to alleviate the psychological burden often experienced by frail HF patients.

A surprising result of this systematic review relies on significant changes in blood markers observed after intervention. In the study of Nagatomi et al. (Nagatomi et al., 2022) serum hemoglobin and blood cholesterol levels significantly increased in the ET group, whereas no changes were found in the control group. Although these results might be controversial, a detailed analysis should be conducted to avoid misunderstanding.

Notably, although these results might be interpreted as increases in blood markers, they probably reflect the maintenance of serum hemoglobin and blood cholesterol values in the ET group. Indeed, blood concentrations remained virtually stable in the ET group, whereas reductions were observed in the control group. These observations might be more indicative of changes in nutritional status in the control group than unexpected increases in the ET group, as the intervention of Nagatomi et al. (Nagatomi et al., 2022) involves nutritional counselling to provide adequate calories and protein intake. Furthermore, the slight increases found in the ET group were within normal ranges for these markers and are likely a product of diet adjustments.

Findings of the present study have clinical implications. The exercise interventions analyzed were generally multicomponent, adaptable, and feasible within routine cardiac rehabilitation settings, suggesting that their implementation does not require highly specialized infrastructure. Given that frail older adults with HF are frequently underrepresented in clinical trials yet commonly encountered in daily clinical care, the observed improvements in functional status, symptoms, and QoL support the integration of structured exercise programs into standard management. Rather than replacing pharmacological therapy, ET should be considered a complementary and patient-centered strategy aimed at preserving independence, reducing functional decline, and potentially lowering healthcare utilization in this high-risk population.

The present study has limitations. First, the relatively small sample size of the included studies limits the generalizability of the findings to the broader population of frail HF patients. Second, while the studies were conducted in multiple countries, the majority were from the United States and Japan, which may restrict the applicability of results to other cultural or healthcare contexts. Third, although the studies employed RCT designs, there was variability in the duration and intensity of ET interventions, making it difficult to draw firm conclusions about the optimal protocols for frail HF patients. Fourth, there was heterogeneity in the methods used to assess frailty and physical performance across studies, potentially introducing bias or inconsistencies in the interpretation of the results. Fifth, the assessment of secondary outcomes, such as QoL and depressive symptoms, varied between studies, and some studies relied on self-reported measures, which are subject to recall bias. Sixth, studies examining strategies ground exclusively on nutritional interventions were not included in the present systematic review. This scenario deserves attention because it might influence the results of the present study (Lorenzo-Lopez et al., 2017; Fard et al., 2019). Hence, this aspect should be examined in future pooled analyses. Seventh, meta-analysis could not be performed due to the variability in outcome measures and intervention protocols across the studies. Eight, no librarian or information specialist was involved in the development of the search strategy. Although the search terms and strategy were carefully designed and discussed among the authors, the absence of a trained librarian may have limited the comprehensiveness and optimization of the search process. The involvement of an information specialist could have further refined the selection of databases, controlled vocabulary (e.g., MeSH terms), and search syntax, potentially increasing the sensitivity and specificity of the search and reducing the risk of missing relevant studies.

Finally, no limitations were set regarding the frailty assessment tools used. This approach was adopted to include as many relevant articles as possible, given that this is the first review addressing this topic. Notably, although the SPPB has demonstrated good accuracy in identifying individuals with frailty (Sanchez-Sanchez et al., 2022), it has not been frequently mentioned in the major guidelines for frailty assessment. Therefore, more studies are needed to confirm the accuracy of the SPPB in identifying frailty, as well as investigations that further validate the results observed with SPPB, such as Symptom-Limited Exercise Tolerance and PA.

## 5 Conclusion

Findings of the present study indicate that cardiac rehabilitation programs based on ET protocols significantly improve health outcomes in frail patients with HF. Specifically, symptomatology of HF, frailty severity, and depressive symptoms were reduced, while performance on multiple physical function tests (i.e., mobility, lower-limb muscle strength, aerobic capacity), PA levels, and QoL were significantly increased. These results highlight the need for additional RCTs to provide further evidence and enable more detailed pooled analyses, which would contribute to a more comprehensive understanding of the benefits of ET in this patient population.

## References

[B1] Afilalo J. Alexander K. P. Mack M. J. Maurer M. S. Green P. Allen L. A. (2014). Frailty assessment in the cardiovascular care of older adults. J. Am. Coll. Cardiol. 63, 747–762. 10.1016/j.jacc.2013.09.070 24291279 PMC4571179

[B2] Agra Bermejo R. M. González Ferreiro R. Varela Román A. Gómez Otero I. Kreidieh O. Conde Sabarís P. (2017). Nutritional status is related to heart failure severity and hospital readmissions in acute heart failure. Int. J. Cardiol. 230, 108–114. 10.1016/J.IJCARD.2016.12.067 28038805

[B3] Altimir S. Lupón J. González B. Prats M. Parajón T. Urrutia A. (2005). Sex and age differences in fragility in a heart failure population. Eur. J. Heart Fail. 7, 798–802. 10.1016/j.ejheart.2004.09.015 16087134

[B4] American College of Sports Medicine (2009). American college of sports medicine position stand. Progression models in resistance training for healthy adults. Med. Sci. Sports Exerc. 41, 687–708. 10.1249/MSS.0b013e3181915670 19204579

[B5] Auyeung T. W. Kwok T. Lee J. Leung P. C. Leung J. Woo J. (2008). Functional decline in cognitive impairment - the relationship between physical and cognitive function. Neuroepidemiology 31, 167–173. 10.1159/000154929 18784415 PMC2824577

[B6] Bai T. Fang F. Li F. Ren Y. Hu J. Cao J. (2020). Sarcopenia is associated with hypertension in older adults: a systematic review and meta-analysis. BMC Geriatr. 20, 279. 10.1186/s12877-020-01672-y 32762638 PMC7409686

[B7] Beeri M. S. Leugrans S. E. Delbono O. Bennett D. A. Buchman A. S. (2021). Sarcopenia is associated with incident Alzheimer’s dementia, mild cognitive impairment, and cognitive decline. J. Am. Geriatr. Soc. 69, 1826–1835. 10.1111/JGS.17206 33954985 PMC8286176

[B8] Chaudhry S. I. Wang Y. Gill T. M. Krumholz H. M. (2010). Geriatric conditions and subsequent mortality in older patients With heart failure. J. Am. Coll. Cardiol. 55, 309–316. 10.1016/j.jacc.2009.07.066 20117435 PMC2832791

[B9] Coelho-Júnior H. J. Gambassi B. B. Irigoyen M.-C. Gonçalves I. D. O. Oliveira P. D. L. L. Schwingel P. A. (2018). Hypertension, sarcopenia, and global cognitive function in community-dwelling older women: a preliminary study. J. Aging Res. 2018, 9758040. 10.1155/2018/9758040 30057815 PMC6051132

[B10] Dai S. Shu D. Meng F. Chen Y. Wang J. Liu X. (2023). Higher risk of sarcopenia in older adults with type 2 diabetes: NHANES 1999–2018. Obes. Facts 16, 237–248. 10.1159/000530241 37011596 PMC10826600

[B11] Darbà J. Ascanio M. Rodríguez A. Charman S. J. Okwose N. C. Stefanetti R. J. (2025). Economic burden of heart failure in Europe: a systematic review of costs and cost-effectiveness. ESC Heart Fail 12, 4055–4068. 10.1002/ehf2.70017 41296502 PMC12719840

[B12] Davies B. Walter S. Rodríguez-Laso A. Carnicero Carreño J. A. García-García F. J. Álvarez-Bustos A. (2022). Differential association of frailty and sarcopenia with mortality and disability: insight supporting clinical subtypes of frailty. J. Am. Med. Dir. Assoc. 23, 1712–1716.e3. 10.1016/J.JAMDA.2022.03.013 35472314

[B13] de Morton N. A. (2009). The PEDro scale is a valid measure of the methodological quality of clinical trials: a demographic study. Aust. J. Physiother. 55, 129–133. 10.1016/S0004-9514(09)70043-1 19463084

[B14] Demant M. N. Gislason G. H. Køber L. Vaag A. Torp-Pedersen C. Andersson C. (2014). Association of heart failure severity with risk of diabetes: a Danish nationwide cohort study. Diabetologia 57 (8), 1595–1600. 10.1007/S00125-014-3259-Z 24849568

[B15] Denfeld Q. E. Winters-Stone K. Mudd J. O. Gelow J. M. Kurdi S. Lee C. S. (2017). The prevalence of frailty in heart failure: a systematic review and meta-analysis. Int. J. Cardiol. 236, 283–289. 10.1016/j.ijcard.2017.01.153 28215466 PMC5392144

[B16] Dobarro D. Costas-Vila A. Melendo-Viu M. Cordeiro-Rodríguez M. Íñiguez-Romo A. Rodríguez-Pascual C. (2023). Home exercise intervention with the vivifrail program in frail older patients with heart failure with reduced ejection fraction. The ExFRAIL-HF randomized trial. Rev. Española Cardiol. (English Ed.) 76, 939–943. 10.1016/j.rec.2023.06.001 37315922

[B17] Fard N. R. P. Amirabdollahian F. Haghighatdoost F. (2019). Dietary patterns and frailty: a systematic review and meta-analysis. Nutr. Rev. 77, 498–513. 10.1093/nutrit/nuz007 31038679

[B18] Fried L. P. Tangen C. M. Walston J. Newman A. B. Hirsch C. Gottdiener J. (2001). Frailty in older adults: evidence for a phenotype. J. Gerontol. - Ser. A Biol. Sci. Med. Sci. 56, M146–M156. 10.1093/gerona/56.3.m146 11253156

[B19] García-Nogueras I. Aranda-Reneo I. Peña-Longobardo L. M. Oliva-Moreno J. Abizanda P. (2017). Use of health resources and healthcare costs associated with frailty: the FRADEA study. J. Nutr. Health Aging 21, 207–214. 10.1007/s12603-016-0727-9 28112778 PMC12879863

[B20] Green S. Higgins J. (2005). Cochrane handbook for systematic reviews of interventions. Available online at: http://www.rismes.it/pdf/Cochrane_handbook2009.rtf (Accessed July 21, 2018).

[B21] Hajek A. Bock J.-O. Saum K.-U. Matschinger H. Brenner H. Holleczek B. (2018). Frailty and healthcare costs-longitudinal results of a prospective cohort study. Age Ageing 47, 233–241. 10.1093/ageing/afx157 29036424

[B22] Hirani V. Blyth F. Naganathan V. Le Couteur D. G. Seibel M. J. Waite L. M. (2015). Sarcopenia is associated with incident disability, institutionalization, and mortality in community-dwelling older men: the Concord health and ageing in men project. J. Am. Med. Dir. Assoc. 16, 607–613. 10.1016/j.jamda.2015.02.006 25820131

[B23] Hoogendijk E. O. Afilalo J. Ensrud K. E. Kowal P. Onder G. Fried L. P. (2019). Frailty: implications for clinical practice and public health. Lancet 394, 1365–1375. 10.1016/S0140-6736(19)31786-6 31609228

[B24] Huang C. Y. Hwang A. C. Liu L. K. Lee W. J. Chen L. Y. Peng L. N. (2016). Association of dynapenia, sarcopenia, and cognitive impairment among community-dwelling older Taiwanese. Rejuvenation Res. 19, 71–78. 10.1089/rej.2015.1710 26165544

[B25] James S. L. Abate D. Abate K. H. Abay S. M. Abbafati C. Abbasi N. (2018). Global, regional, and national incidence, prevalence, and years lived with disability for 354 diseases and injuries for 195 countries and territories, 1990-2017: a systematic analysis for the global burden of disease study 2017. Lancet 392, 1789–1858. 10.1016/S0140-6736(18)32279-7 30496104 PMC6227754

[B26] Janssen I. Shepard D. S. Katzmarzyk P. T. Roubenoff R. (2004). The healthcare costs of sarcopenia in the United States. J. Am. Geriatr. Soc. 52, 80–85. 10.1111/J.1532-5415.2004.52014.X 14687319

[B27] Jordan C. Charman S. J. Batterham A. M. Flynn D. Houghton D. Errington L. (2023). Habitual physical activity levels of adults with heart failure: systematic review and meta-analysis. Heart 109, 1357–1362. 10.1136/HEARTJNL-2022-321943 36849238 PMC10511969

[B28] Kato A. (2013). Muscle wasting is associated with reduced exercise capacity and advanced disease in patients with chronic heart failure. Future Cardiol. 9, 767–770. 10.2217/fca.13.74 24180533

[B29] Khan M. S. Shahid I. Bennis A. Rakisheva A. Metra M. Butler J. (2024). Global epidemiology of heart failure. Nat. Rev. Cardiol. 21, 717–734. 10.1038/s41569-024-01046-6 38926611

[B30] Kojima G. (2016). Frailty as a predictor of hospitalisation among community-dwelling older people: a systematic review and meta-analysis. J. Epidemiol. Commun. Health (1978) 70, 722–729. 10.1136/jech-2015-206978 26933121

[B31] Kojima G. (2017a). Frailty as a predictor of disabilities among community-dwelling older people: a systematic review and meta-analysis. Disabil. Rehabil. 39, 1897–1908. 10.1080/09638288.2016.1212282 27558741

[B32] Kojima G. (2017b). Frailty significantly increases the risk of fractures among middle-aged and older people. Evid. Based Nurs. 20, 119–120. 10.1136/eb-2017-102769 28847777

[B33] Kojima G. (2018a). Frailty as a predictor of nursing home placement among community-dwelling older adults. J. Geriatric Phys. Ther. 41, 42–48. 10.1519/JPT.0000000000000097 27341327

[B34] Kojima G. (2018b). Quick and simple FRAIL scale predicts incident activities of daily living (ADL) and instrumental ADL (IADL) disabilities: a systematic review and meta-analysis. J. Am. Med. Dir. Assoc. 19, 1063–1068. 10.1016/j.jamda.2018.07.019 30206033

[B35] Kraemer W. J. Ratamess N. A. (2004). Fundamentals of resistance training: progression and exercise prescription. Med. Sci. Sports Exerc. 36, 674–688. 10.1249/01.MSS.0000121945.36635.61 15064596

[B36] LaMonte M. J. (2018). Physical activity and heart failure: taking steps to control a major public health burden. Am. J. Lifestyle Med. 14, 555–570. 10.1177/1559827618769609 33110401 PMC7566185

[B37] Lavie C. J. De Schutter A. Alpert M. A. Mehra M. R. Milani R. V. Ventura H. O. (2014). Obesity paradox, cachexia, frailty, and heart failure. Heart Fail. Clin. 10, 319–326. 10.1016/j.hfc.2013.12.002 24656108

[B38] Liang K. V. Pike F. Argyropoulos C. Weissfeld L. Teuteberg J. Dew M. A. (2011). Heart failure severity scoring system and Medical- and health-related quality-of-life outcomes: the HEMO study. Am. J. Kidney Dis. 58, 84–92. 10.1053/J.AJKD.2011.01.029 21549465 PMC4508008

[B39] Liberati A. Altman D. G. Tetzlaff J. Mulrow C. Gøtzsche P. C. Ioannidis J. P. A. (2009). The PRISMA statement for reporting systematic reviews and meta-analyses of studies that evaluate health care interventions: explanation and elaboration. PLoS Med. 6, e1000100. 10.1371/journal.pmed.1000100 19621070 PMC2707010

[B40] Lorenzo-Lopez L. Maseda A. de Labra C. Regueiro-Folgueira L. Rodriguez-Villamil J. L. Millan-Calenti J. C. (2017). Nutritional determinants of frailty in older adults: a systematic review. BMC Geriatr. 17, 108. 10.1186/s12877-017-0496-2 28506216 PMC5433026

[B41] Lupón J. González B. Santaeugenia S. Altimir S. Urrutia A. Más D. (2008). Prognostic implication of frailty and depressive symptoms in an outpatient population with heart failure. Rev. Española Cardiol. (English Ed.) 61, 835–842. 10.1016/s1885-5857(08)60231-5 18684366

[B42] McNallan S. M. Chamberlain A. M. Gerber Y. Singh M. Kane R. L. Weston S. A. (2013). Measuring frailty in heart failure: a community perspective. Am. Heart J. 166, 768–774. 10.1016/j.ahj.2013.07.008 24093859 PMC3841984

[B43] Morley J. E. Vellas B. Abellan van Kan G. Anker S. D. Bauer J. M. Bernabei R. (2013). Frailty consensus: a call to action. J. Am. Med. Dir. Assoc. 14, 392–397. 10.1016/j.jamda.2013.03.022 23764209 PMC4084863

[B44] Mudge A. M. Pelecanos A. Adsett J. A. (2021). Frailty implications for exercise participation and outcomes in patients with heart failure. J. Am. Geriatr. Soc. 69, 2476–2485. 10.1111/JGS.17145 33826158

[B45] Nagata C. de A. Garcia P. A. Hamu T. C. D. da S. Caetano M. B. D. Costa R. R. Leal J. C. (2023). Are dose-response relationships of resistance training reliable to improve functional performance in frail and pre-frail older adults? A systematic review with meta-analysis and meta-regression of randomized controlled trials. Ageing Res. Rev. 91, 102079. 10.1016/j.arr.2023.102079 37774931

[B46] Nagatomi Y. Ide T. Higuchi T. Nezu T. Fujino T. Tohyama T. (2022). Home‐based cardiac rehabilitation using information and communication technology for heart failure patients with frailty. ESC Heart Fail 9, 2407–2418. 10.1002/EHF2.13934 35534907 PMC9288767

[B47] Pandey A. Kitzman D. Reeves G. (2019). Frailty is intertwined with heart failure: mechanisms, prevalence, prognosis, assessment, and management. JACC Heart Fail 7, 1001–1011. 10.1016/j.jchf.2019.10.005 31779921 PMC7098068

[B48] Pandey A. Kitzman D. W. Nelson M. B. Pastva A. M. Duncan P. Whellan D. J. (2023). Frailty and effects of a multidomain physical rehabilitation intervention among older patients hospitalized for acute heart failure: a secondary analysis of a randomized clinical trial. JAMA Cardiol. 8, 167–176. 10.1001/JAMACARDIO.2022.4903 36598761 PMC9857661

[B49] Papathanasiou J. V. (2020). Are the group-based interventions improving the functional exercise capacity and quality of life of frail subjects with chronic heart failure? J. Frailty Sarcopenia Falls 5, 102–108. 10.22540/JFSF-05-102 33283076 PMC7711733

[B50] Prokopidis K. Nortcliffe A. Okoye C. Venturelli M. Lip G. Y. H. Isanejad M. (2025). Length of stay and prior heart failure admission in frailty and heart failure: a systematic review and meta-analysis. ESC Heart Fail 12, 2417–2426. 10.1002/ehf2.15300 40205981 PMC12287781

[B51] Rodríguez-Mañas L. Féart C. Mann G. Viña J. Chatterji S. Chodzko-Zajko W. (2013). Searching for an operational definition of frailty: a delphi method based consensus statement. The frailty operative definition-consensus conference project. J. Gerontol. - Ser. A Biol. Sci. Med. Sci. 68, 62–67. 10.1093/gerona/gls119 22511289 PMC3598366

[B52] Salomon J. A. Wang H. Freeman M. K. Vos T. Flaxman A. D. Lopez A. D. (2012). Healthy life expectancy for 187 countries, 1990-2010: a systematic analysis for the global burden disease study 2010. Lancet 380, 2144–2162. 10.1016/S0140-6736(12)61690-0 23245606

[B53] Sanchez-Sanchez J. L. Carnicero-Carreño J. A. Garcia-Garcia F. J. Álvarez-Bustos A. Rodríguez-Sánchez B. Rodríguez-Mañas L. (2022). Physical performance measures in frailty screening: diagnostic and prognostic accuracy in the Toledo study of healthy ageing. Maturitas 165, 18–25. 10.1016/j.maturitas.2022.07.004 35849911

[B54] Shuvy M. Zwas D. R. Lotan C. Keren A. Gotsman I. (2020). Albuminuria: associated with heart failure severity and impaired clinical outcomes. Can. J. Cardiol. 36, 527–534. 10.1016/J.CJCA.2019.09.001 31926740

[B55] Springer J. Springer J. I. Anker S. D. (2017). Muscle wasting and sarcopenia in heart failure and beyond: update 2017. ESC Heart Fail 4, 492–498. 10.1002/ehf2.12237 29154428 PMC5695190

[B56] Studenski S. Perera S. Patel K. Rosano C. Faulkner K. Inzitari M. (2011). Gait speed and survival in older adults. JAMA 305, 50–58. 10.1001/jama.2010.1923 21205966 PMC3080184

[B57] Suzuki T. Palus S. Springer J. (2018). Skeletal muscle wasting in chronic heart failure. ESC Heart Fail 5, 1099–1107. 10.1002/ehf2.12387 30548178 PMC6300810

[B58] Veronese N. Sigeirsdottir K. Eiriksdottir G. Marques E. A. Chalhoub D. Phillips C. L. (2017). Frailty and risk of cardiovascular diseases in older persons: the age, gene/environment susceptibility-reykjavik study. Rejuvenation Res. 20, 517–524. 10.1089/rej.2016.1905 28602121 PMC5731544

[B59] Vidán M. T. Blaya-Novakova V. Sánchez E. Ortiz J. Serra-Rexach J. A. Bueno H. (2016). Prevalence and prognostic impact of frailty and its components in non-dependent elderly patients with heart failure. Eur. J. Heart Fail. 18, 869–875. 10.1002/ejhf.518 27072307

[B60] Witham M. D. Gray J. M. Argo I. S. Johnston D. W. Struthers A. D. McMurdo M. E. T. (2005). Effect of a seated exercise program to improve physical function and health status in frail patients ≥70 years of age with heart failure. Am. J. Cardiol. 95, 1120–1124. 10.1016/j.amjcard.2005.01.031 15842989

[B61] Yang X. Lupón J. Vidán M. T. Ferguson C. Gastelurrutia P. Newton P. J. (2018). Impact of frailty on mortality and hospitalization in chronic heart failure: a systematic review and meta-analysis. J. Am. Heart Assoc. 7, e008251. 10.1161/JAHA.117.008251 30571603 PMC6405567

[B62] Yang X. Li S. Xu L. Liu H. Li Y. Song X. (2024). Effects of multicomponent exercise on frailty status and physical function in frail older adults: a meta-analysis and systematic review. Exp. Gerontol. 197, 112604. 10.1016/J.EXGER.2024.112604 39426607

[B63] Yin J. Lu X. Qian Z. Xu W. Zhou X. (2019). New insights into the pathogenesis and treatment of sarcopenia in chronic heart failure. Theranostics 9, 4019–4029. 10.7150/thno.33000 31281529 PMC6592172

[B64] Zhang Y. Zhang J. Ni W. Yuan X. Zhang H. Li P. (2021). Sarcopenia in heart failure: a systematic review and meta-analysis. ESC Heart Fail 8, 1007–1017. 10.1002/EHF2.13255 33576177 PMC8006658

